# Allergic response to medical products in patients with alpha-gal syndrome

**DOI:** 10.1016/j.jtcvs.2021.03.100

**Published:** 2021-04-09

**Authors:** Kasinath V. Kuravi, Lori T. Sorrells, Joseph R. Nellis, Farzana Rahman, Anneke H. Walters, Robert G. Matheny, Shailesh K. Choudhary, David L. Ayares, Scott P. Commins, John R. Bianchi, Joseph W. Turek

**Affiliations:** a Revivicor Incorporated, United Therapeutics, Blacksburg, Va; b Duke Congenital Heart Surgery Research and Training Laboratory, Durham, NC; c CorMatrix Cardiovascular Inc, Roswell, Ga; d Division of Allergy, Immunology, and Rheumatology, University of North Carolina, Chapel Hill, NC; e Division of Cardiovascular and Thoracic Surgery, Department of Surgery, Duke University, Durham, NC.

**Keywords:** alpha-gal, alpha-gal syndrome, allergy, bioprosthetic valve, chronic inflammation, early valve degradation, coronary artery disease

## Abstract

**Background:**

Galactose-*α*-1,3-galactose (alpha-gal) is a carbohydrate that is ubiquitously expressed in all mammals except for primates and humans. Patients can become sensitized to this antigen and develop alpha-gal syndrome (AGS), or a red meat allergy. Symptoms range from generalized gastroenteritis and malaise to anaphylaxis, and in endemic areas, the prevalence can be as high as 20%. Although AGS patients commonly avoid alpha-gal by avoiding meat, patients have also developed symptoms due to animal-derived medical products and devices. With the rise in transcatheter aortic valve replacement, we investigate the immunogenicity of common cardiac materials and valves.

**Objective:**

To assess the in vitro immunoglobulin E response toward common medical products, including cardiac patch materials and bioprosthetic valves in patients with AGS.

**Methods:**

Immunoblot and immunohistochemistry techniques were applied to assess immunoglobulin E reactivity to various mammalian derived tissues and medical products for patients with AGS.

**Results:**

AGS serum showed strong reactivity to all of the commercially available, nonhuman products tested, including various decellularized cardiac patch materials and bioprosthetic aortic valves. AGS serum did not react to tissues prepared using alpha-gal knockout pigs.

**Conclusions:**

Despite commercial decellularization processes, alpha-gal continues to be present in animal-derived medical products, including bioprosthetic valves. Serum from patients with AGS demonstrates a strong affinity for these products in vitro. This may have serious potential implications for sensitized patients undergoing cardiac surgery, including early valve failure and accelerated coronary artery disease.

## Graphical Abstract

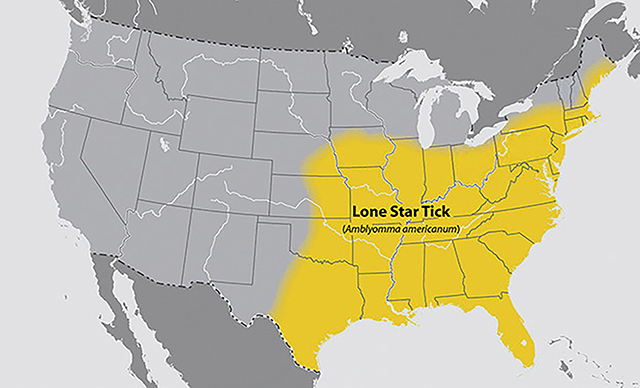

The Lone Star tick generates alpha-gal IgE, which binds commercial bioprosthetic valves.

Galactose-*α*-1,3-galactose (alpha-gal) is a cell membrane carbohydrate that is present in all mammals, except for humans and old-world primates who lack a functional glycoprotein galactosyltransferase alpha-1,3 (*GGTA1*) gene.^1^ Alpha-gal modifies extracellular proteins and lipids, and is ubiquitously expressed across all organs, tissues, and cell types to varying degrees in lesser mammals. Humans uniformly develop immunoglobulin (Ig) A, IgM, and IgG serotypes to the antigen due to its presence in the gut microbiome.^1,2^ An IgE, or allergic, response to the antigen was not documented until 2008 when patients in the southeastern United States were noted to be experiencing a disproportionate number of anaphylactic episodes following the administration of cetuximab (a chimeric monoclonal murine-human antibody).^3^ Subsequent work went on to demonstrate an association between *Amblyomma americanum* tick bites and alpha-gal IgE seroconversion within the United States through an unknown mechanism.^4^ Internationally, similar relationships were seen with *Ixodes holocyclus* and *Ixodes ricinus* in Australia and Europe, respectively, affecting approximately 20% of patients in endemic regions.^5,6^ Following exposure to alpha-gal, and animal-derived products, including red meat, patients can experience symptoms such as urticaria, angioedema, and anaphylaxis. In fact, within specific geographic areas anaphylaxis due to alpha-gal IgE seroconversion is more common than all other forms of dietary anaphylaxis combined.^4,7^ Although a majority of patients knowingly or unknowingly avoid these products, others continue to experience symptoms and eventually diagnosed with alpha-gal syndrome (AGS), a diagnosis based on clinical symptoms and alpha-gal IgE >0.1 IU/mL.

AGS is an underreported and often unrecognized allergic condition. Case reports estimate the prevalence of AGS to be between 8% and 46%, with the highest rates in endemic regions such as the southeastern United States.^4,8–12^ Although high, the numbers are likely higher because many patients experience vague, transient symptoms that do not require medical attention and often have an identifiable dietary trigger (eg, red meat, dairy products, or other animal-derived products).^4^ Additionally, the average time between symptoms onset and diagnosis is 7 years—highlighting the chronicity of this condition, low awareness of it within the medical community, and the number of patients who once asked about their symptoms and who have since given up.^13–16^ Although AGS may appear benign, particularly for patients with vague symptoms, the long-term effects of chronic inflammation due to animal-derived supplements or surgical implants may be devastating.

Alpha-gal has always been an issue in cardiac surgery, despite the paucity of literature on the topic. Alpha-gal is among the 3 critical antigens in xenotransplantation, and its immunogenicity in humans is well established.^17,18^ Decellularization processes and glutaraldehyde fixation slow the immune response against commercially available bioprosthetic valves, but they do not stop it.^19–21^ To this point, all patients receiving bioprosthetic valves experience a rise in alpha-gal IgG and IgM.^20,21^ Younger patients display stronger immune responses, and it follows that age is a risk factor for early valve degradation following bioprosthetic valve replacement.^22–24^ These data have generated criticism regarding the ongoing use of bioprosthetic valves and the rise in transcatheter aortic valve replacement, motivating some to develop alternative models and processes.^25–28^ Even less is known about the long-term clinical implications for patients with AGS, particularly after receiving a bioprosthetic valve replacement.

Reports suggest an association between AGS, delayed anaphylaxis, and accelerated valve degradation, although the literature is limited on the implications of recently discovered IgE serotypes.^8,29–32^ Therefore in this study, we set out to evaluate the immunogenicity of AGS sera on commercially available supplements and surgical implants, including cardiac patch materials and bioprosthetic valves. These data were then compared with tissues from GGTA1^−/−^ pigs, who lack the alpha-gal antigen, as a potential solution to alpha-gal mediated antigenicity.

## MATERIALS AND METHODS

### Animals

Five wild-type (WT) pigs, 5 GGTA1^−/−^ pigs, and 1 GGTA1^+/−^ pig were obtained from Revivicor Inc (Blacksburg, Va). GGTA1^+/−^ represents an alpha-gal knockdown pig, and GGTA1^−/−^ represents an alpha-gal knockout (GalSafe) pig.^33^ All animals were euthanized and processed under Institutional Animal Care and Use Committee reviewed protocols.

### Commercially Available Medications

Common animal-derived medications were prepared and analyzed for the presence of alpha-gal using antibody and human sera-based immunoblot analysis. A dose of 220 mg Armour Thyroid (Allergan, Irvine, Calif), a porcine thyroxine and triiodothyronine prescription, was crushed with mortar and pestle, mixed in 4 mL phosphate-buffered saline (PBS) with protease inhibitors, and subsequently aliquoted (100 *μ*L) for immunoblot analysis.

Preparations of 25 mg and 40 mg Zenpep (Allergan), a porcine-derived pancreatic insufficiency medication composed of proteases, lipases, and amylases, were mixed in 0.5 mL PBS with protease inhibitors. A dose of 50 mg Pancreatin (Now Foods, Bloomingdale, Ill), an over-the-counter dietary aid, was mixed with 0.5 mL of PBS with protease inhibitors. All samples were centrifuged at 5000× gravity for 5 minutes at 4°C, before 100 *μ*L supernatant was aliquoted and stored.

An amount of 50 mg EnteraGam (Entera Health, Cary, NC), a bovine immunoglobulin and immunoprotein isolate intended for the management of irritable bowel syndrome and inflammatory bowel disease, was dissolved in 1 mL PBS with protease inhibitors. A total of 25 *μ*L working stock was further diluted into 100 *μ*L aliquots.

An amount of 100 mg Gelatine (Knox Company, Parsipanny, NJ) and 100 mg laboratory-grade gelatin (104078; MilliporeSigma, St Louis, Mo), porcine skin, and bone derivatives, were individually dissolved in 0.5 mL PBS and heated at 55°C for 15 minutes. A total of 5 *μ*L of each mixture was then resuspended in 95 *μ*L PBS. Samples were heated again at 55°C for 5 minutes.

An amount of 20 mg porcine thyroglobulin (T1126; MilliporeSigma), a known alpha-gal epitope-carrying positive control, was dissolved in 0.5 mL PBS with protease inhibitors.

### Commercially Available Surgical Implants

Surgical materials tested included Cardiocel acellular collagen scaffold (LeMaitre Vascular, Burlington, Mass), Photofix decellularized bovine pericardium (Cryolife, Kennesaw, Ga), first- and second-generation Cor PATCH acellular porcine small intestinal submucosa (CorMatrix Cardiovascular Inc, Sunnyvale, Calif), ProCol bovine mesenteric vein (LeMaitre Vascular), Epic Supra porcine bioprosthetic aortic valve; Abbott, Abbott Park, Ill), Perimount Magna Ease (bovine bioprosthetic aortic valve (Edwards Lifesciences, Irvine, Calif), and CryoValve SG pulmonary valve decellularized human pulmonary valve conduit (Cryolife).

### Blood Collection and Sample Preparation

Whole blood was collected from human volunteers at the University of North Carolina, after informed consent was obtained from our institutional review board (#16–1533). Samples were allowed to clot at room temperature, before the serum was separated by centrifuge at 2000× gravity, isolated, and stored at −80°C. Samples provided for the study include 15 patients with AGS, 15 patients without AGS (controls), and 1 patient without clinical symptoms but with elevated alpha-gal IgE (sensitized). The diagnosis of AGS was established before enrollment in the study on the basis of clinical symptoms (eg, gastroenteritis, hives, pruritus, angioedema, hypotension, or airway compromise) following meat consumption with an alpha-gal IgE titer >0.35 IU/mL. The control patients had no signs or symptoms with the consumption of meat and alpha-gal IgE titers <0.1 IU/mL.

Alpha-gal IgE levels were measured by a modification of the Immuno-CAP assay using biotinylated alpha-gal on the solid phase (Phadia ThermoFisher, Portage, Mich).^3^ Laboratory testing has been validated and is commercially available through ViraCor-Eurofins Laboratories (Lee’s Summit, Mo).

Due to the limited supply of AGS patient sera, we could not test each patient’s sera with every tissue or material included in the study. All assays were run in duplicates to avoid isolated findings.

### Immunohistochemistry

Immunohistochemistry was carried out across various formalin-fixed, paraffin-embedded tissues including WT and GGTA1^−/−^ pig organs as well as the commercially available cardiac surgery materials. Samples were sectioned at 4 *μ*m, air-dried, and deparaffinized. Endogenous peroxidases were quenched using Dual Endogenous Enzyme Block (S2003; Agilent Technologies, Santa Clara, Calif) for 10 minutes. After washing with Tris-Buffered Saline with Tween (TBST) buffer (T9039; MilliporeSigma), sections were blocked for endogenous avidin and biotin with an Avidin/Biotin Blocking Kit (004303; Invitrogen, Carlsbad, Calif).

*Griffonia simplicifolia* lectin I-isolectin B4 (GSL I-B_4_) (B-1205; Vector Laboratories, Burlingame, Calif) binds alpha-galactose residues and was utilized to qualitatively identify the presence of alpha-gal antigen within the specimens. Briefly, 100 *μ*L GSL I-B_4_, diluted at 1:100 in antibody diluent (S0809; Agilent Technologies), was dispensed onto each slide and allowed to incubate at room temperature for 30 minutes. After incubation, slides were rinsed in TBST and incubated in 100 *μ*L Vectastain ABC Reagent (PK-6105; Vector Laboratories) for 30 minutes. Slides were then rinsed in TBST. GSL I-B_4_ binding was detected with a 5-minute incubation in 100 *μ*L diaminobenzidine tetrahydrochloride (K346811–2; Agilent Technologies). Slides were rinsed in tap water, counterstained with hematoxylin (3801575; VWR Inc, Radnor, Pa), mounted with glass coverslips, and imaged.

A similar process was used to detect serum IgE binding to the prepared specimens. Briefly, endogenous peroxidases were quenched using Dual Edogenous Enzyme Block for 10 minutes and rinsed before the addition of 200 *μ*L of either AGS patient or control serum. Slides were incubated overnight at 4°C. Slides were rinsed in TBST and then incubated with 100 *μ*L mouse antihuman IgE Fc (99806; Abcam, Cambridge, Mass) diluted at 1:1000 for 30 minutes. Slides were again rinsed in TBST, and incubated with diaminobenzidine tetrahydrochloride for 5 minutes. After being rinsed in tap water, the slides were counterstained with hematoxylin, and mounted with glass coverslips. Each experiment was performed twice to confirm the results.

### Immunoblot

Immunoblot was carried out across WT, GGTA1^+/−^ and GGTA1^−/−^ pig organs, and the previously listed medications and cardiac surgery implants. Porcine tissue samples were homogenized using Tissue Protein Extraction Reagent (78510; ThermoFisher, Waltham, Mass) with protease inhibitors in a bead beater. Tissue lysates were obtained by centrifuging samples at 5000× gravity for 5 minutes, leaving behind the tissue debris. Protein concentrations were obtained using a bicinchonic acid protein assay kit (23225; ThermoFisher). An amount of 10 *μ*g protein per lane was loaded in Criterion TGX Precast sodium dodecyl sulfate polyacrylamide gel electrophoresis gels (Bio-Rad, Hercules, Calif).

Surgical implants were prepared in a similar manner. Briefly, 60 mg ProCol graft, 50 mg Epi Supra valve, 60 mg Perimount Magna Ease valve, and 90 mg CryoValve SG Pulmonary Valve conduit were homogenized in 500 *μ*L tissue protein extraction reagent with protease inhibitors in a bead beater. Lysates were mixed with sample buffer in a 1:1 ratio. Surgical patch materials were prepared by vortexing the materials in their respective sample buffers for 2 hours (150 mg Photofix/375 *μ*L buffer, 100 mg Cardiocel/350 *μ*L buffer, 40 mg first- and second-generation Cor Patch/350 *μ*L buffer). An amount of 40 *μ*L of each commercially available product, including prepared medications, were loaded into their respective lanes.

Polyacrylamide gel electrophoresis was performed under reducing conditions and developed in standard fashion. Proteins were transferred to nitrocellulose membranes using the Trans-Turbo Blotting System (Bio-Rad). Membranes were stained with Swift Membrane Stain (786–677; G-Bioscience, St Louis, Mo) for total protein detection, per the manufacturer’s instructions. Pierce Protein-Free Block Buffer (37572; ThermoFisher) was used as a membrane blocker and antibody diluent.

The presence of alpha-gal was measured by incubating membranes with the mouse monoclonal anti-alpha-gal antibody (ie, M86) (ALX-801–090-1; Enzo Life Sciences, Farmingdale, NY) at 1:250 dilution overnight at 4°C. Goat anti-mouse IgM mu chain (98679; Abcam) secondary antibody was used at 1:25000 dilution for detection.

Human IgE immunoblotting was done by incubating 1 mL AGS serum, sensitized serum, or control serum with a nitrocellulose membrane overnight at 4°C. Human IgE/alpha-gal glycosylated protein complexes were detected by mouse anti-human IgE Fc (99806; Abcam). The membranes were washed with PBST buffer and incubated with a Clarity Western Enhanced Chemiluminescence Substrate (1705060S; Bio-Rad) per the manufacturer’s recommendations and scanned on a FluoroChem R Imager (Protein Simple, San Jose, Calif). Each experiment was performed twice to confirm the results.

## RESULTS

### Alpha-Gal Glycosylation Profile of Porcine Organ and Tissue Extracts

**FIGURE E1.**
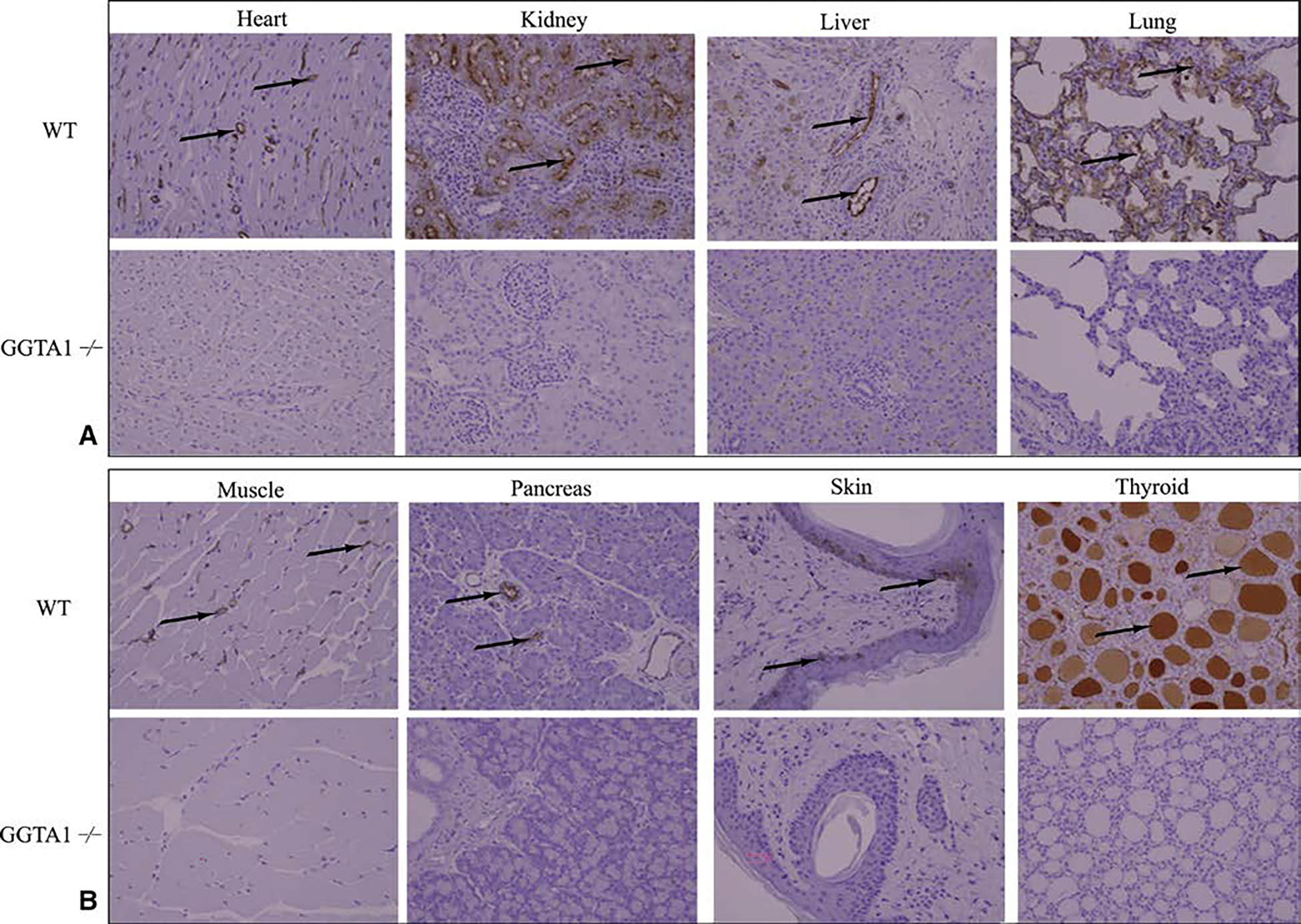
Alpha-gal immunohistochemistry for wild type (WT) and glycoprotein galactosyltransferase *α*-1,3 (GGTA1^−/−^) porcine organs. A and B, WT and GGTA1^−/−^ tissues were stained with biotinylated griffonia simplicifolia lectin I – isolectin B_4_ (GSL I-B_4_) lectin to demonstrate the presence or absence of alpha-gal. The presence of brown pigment (*black arrows*) indicates a positive reaction with the alpha-gal epitope in wild type tissues. The lack of brown pigment confirms absence of alpha-gal in glycoprotein galactosyltransferase *α*-1,3 (GGTA1^−/−^) pig tissues. All images are at 200×.

**FIGURE E2.**
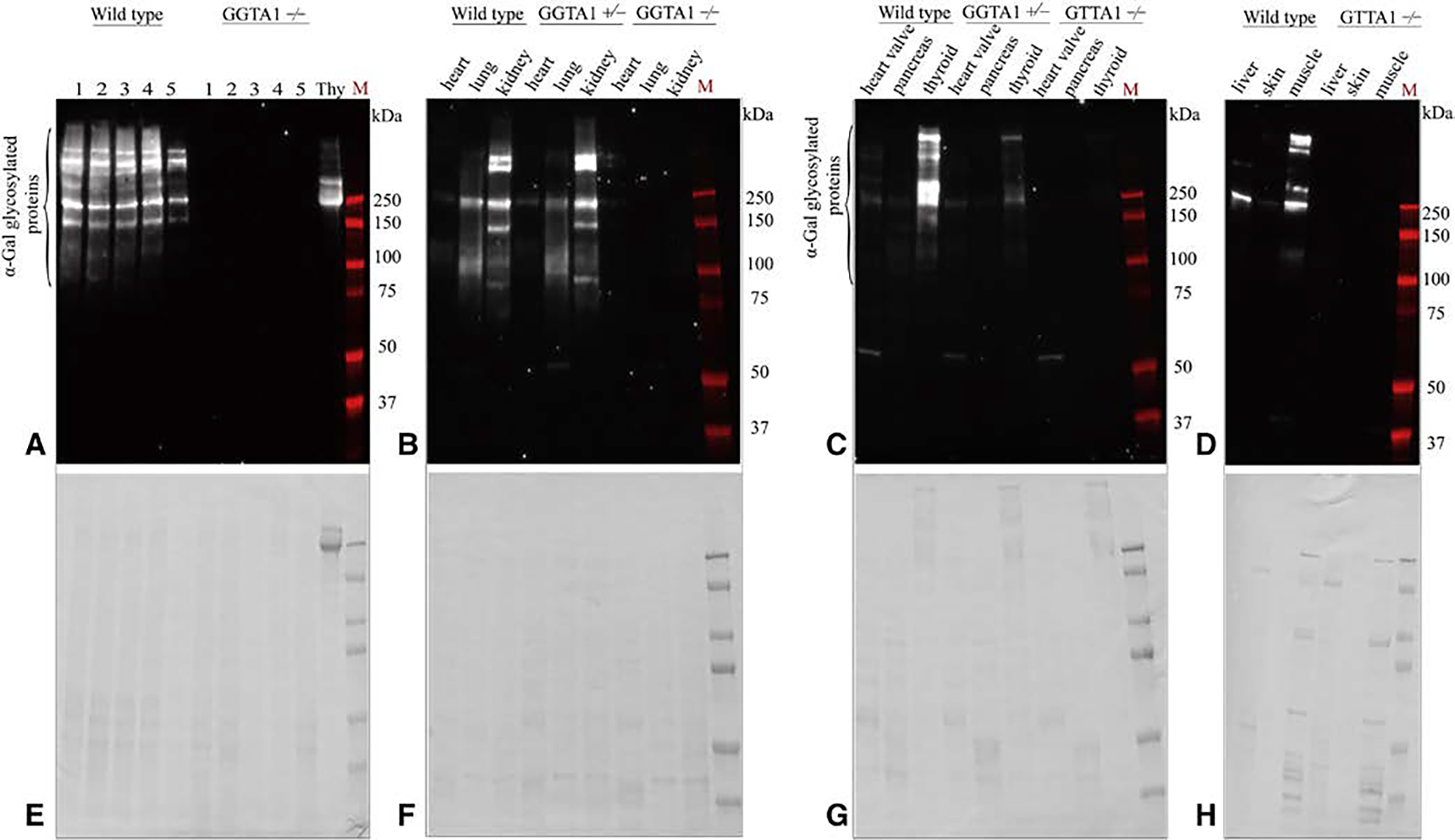
Immunoblot analysis of alpha-gal glycosylated proteins in different porcine organs. A, Protein extracts from wild type (WT) and glycoprotein galactosyltransferase *α*-1,3 (GGTA1^−/−^) pig kidney samples were detected using alpha-gal epitope binding antibody (ie, M86). B and C, Alpha-gal binding profiles of WT, GGTA1^+/−^, and GGTA1^−/−^ porcine heart, lung, kidney, heart valve, pancreas, and thyroid tissue samples using alpha-gal syndrome (AGS) patient serum. D, M86 detection of WT and GGTA1^−/−^ liver, skin and muscle extracts. E though H, The Coomassie stains for the associated immunoblots, indicating the presence of protein. Positive signal in WT and GGTA1^+/−^ samples indicates presence of alpha-gal glycosylation. No signal in GGTA1^−/−^ samples indicates absence of alpha-gal glycosylation.

Immunohistochemistry using GSL I-B_4_, a lectin that binds alpha-galactose residues, confirmed the presence of alpha-gal in all WT pig samples to varying degrees (Figure E1). In the kidney, alpha-gal is concentrated within the glomeruli, whereas in the thyroid, it is predominately seen in the colloid and perifollicular vessels. WT pancreas, heart, skeletal muscle, and skin predominately show alpha-gal concentrated within the vasculature and in the case of skin, the basement membrane as well (Figure E1). All WT immunoblot porcine tissue preparations demonstrated M86 staining between 90 kD and 250 kD, to varying degrees, indicating the presence of alpha-gal glycosylated proteins (Figure E2). Alpha-gal expression was the highest in thyroid and kidney, with proteins predominately running at high molecular weights.

M86 was sensitive enough to detect an intermediate alpha-gal signal in GGTA1^+/−^ knockdown pigs and no signal in knockout (ie, GGTA1^−/−^) pigs. Total protein staining in the assays confirmed the absence of alpha-gal in the GGTA1^−/−^ pig model (Figure E2).

### Human Alpha-Gal IgE Binding in Porcine Organ and Tissue Extracts

**FIGURE E3.**
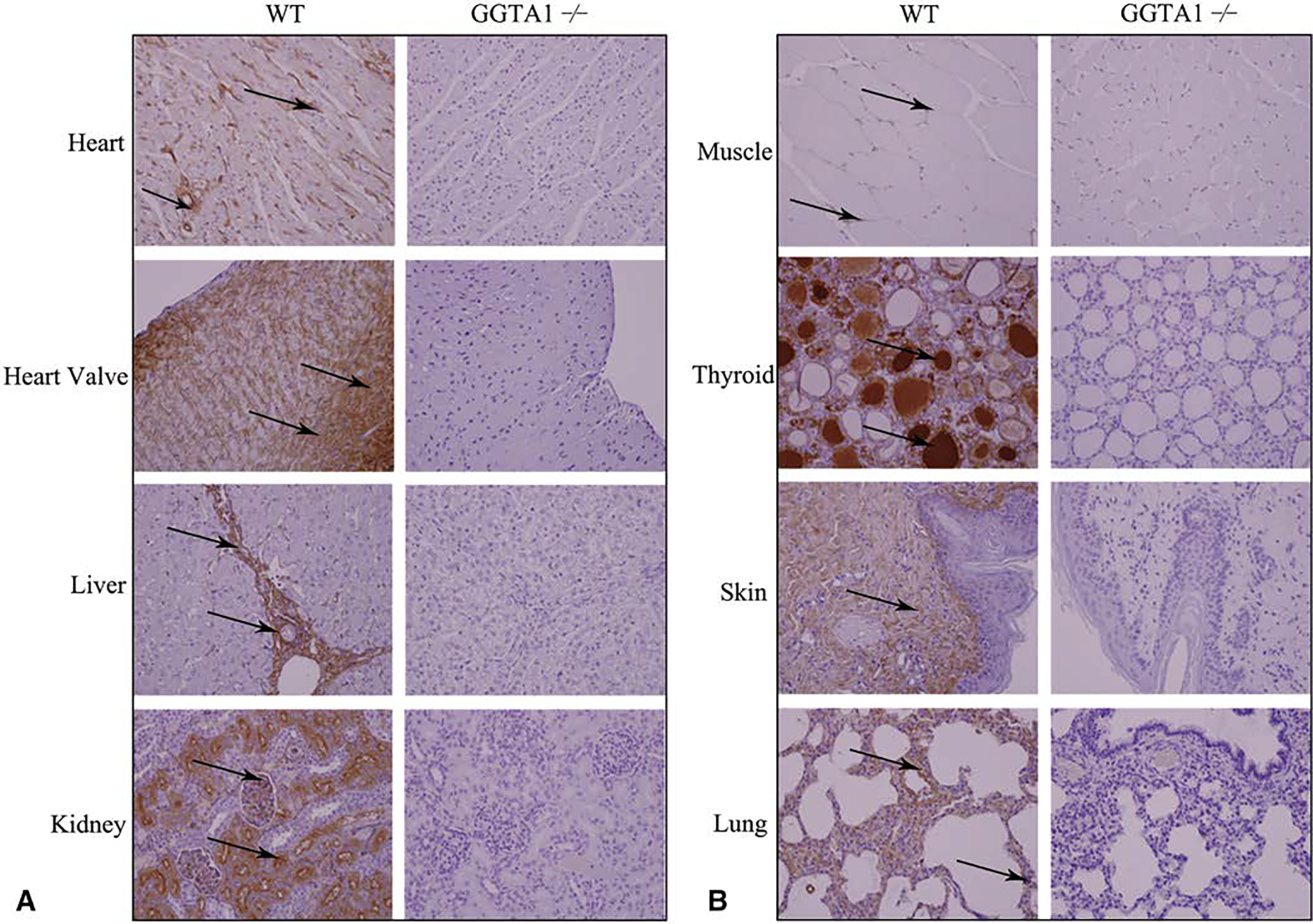
Alpha-gal immunoglobulin E (IgE) reactivity toward glycosylated proteins in different wild type (WT) and glycoprotein galactosyltransferase *α*-1,3 (GGTA1^−/−^) porcine organs. A and B, Alpha-gal binding profiles of WT and GGTA1^−/−^ porcine heart, heart valve, liver, kidney, muscle, thyroid, skin, and lung using alpha-gal syndrome (AGS) patient sera. Positive signal (*brown*) represents the presence of alpha-gal IgE binding. All images are at 200×.

**FIGURE E4.**
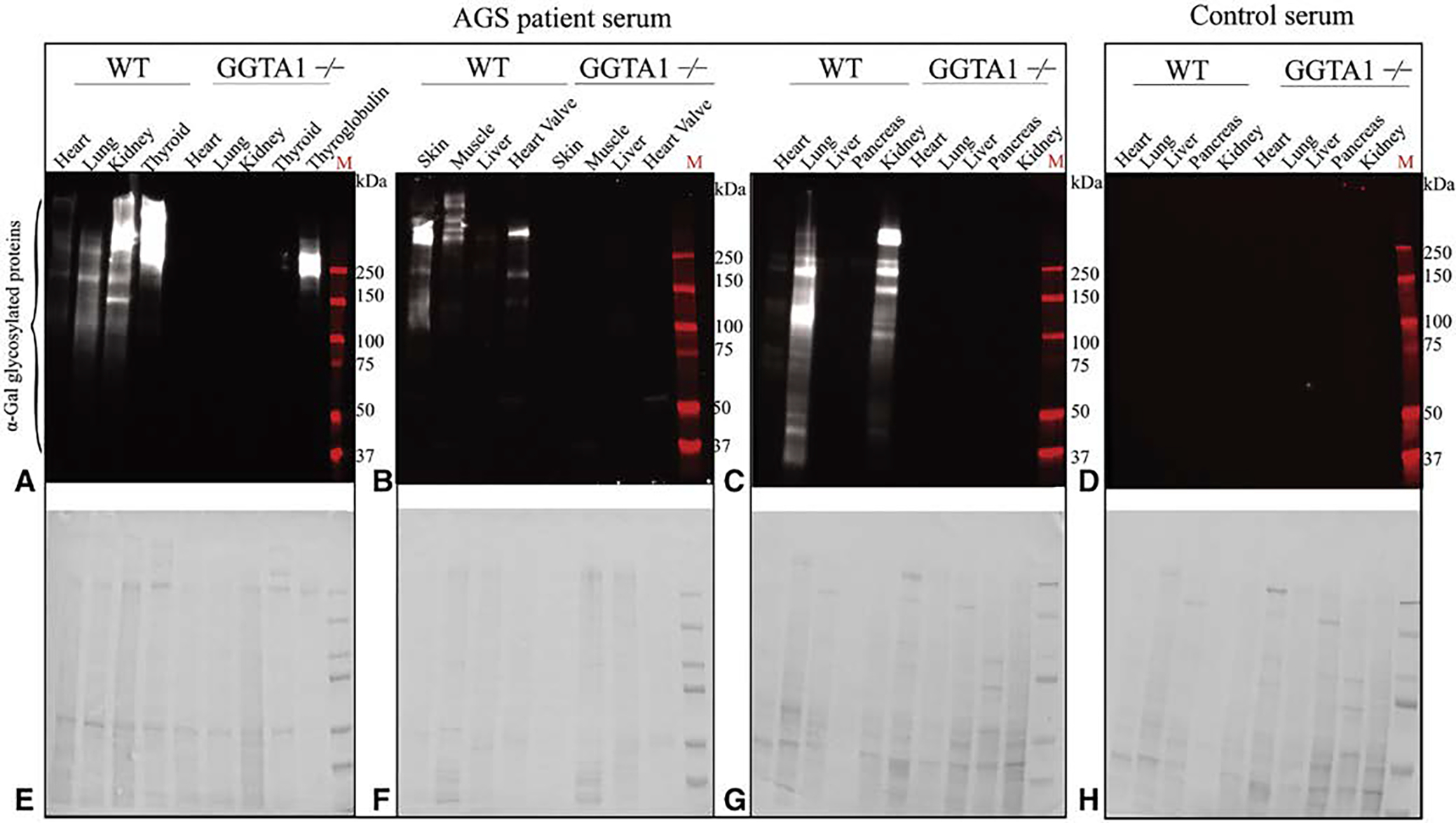
Alpha-gal immunoglobulin E (IgE) reactivity toward glycosylated proteins in different wild type (WT) and glycoprotein galactosyltransferase *α*-1,3 (GGTA1^−/−^) porcine organs. A through C, Alpha-gal binding profiles of WT and GGTA1^−/−^ porcine heart, lung, kidney, thyroid, skin, muscle, liver, heart, heart valve, and pancreas using alpha-gal syndrome (AGS) patient serum. Positive signal represents the presence of alpha-gal glycosylated proteins. D, Alpha-gal binding profiles of WT and GGTA1^−/−^ heart, lung, liver, pancreas, and kidney using control serum from patients without AGS. No signal is seen representing the absence of alpha-gal glycosylated proteins. E though H, The Coomassie stains for the associated immunoblots, indicating the presence of protein.

**FIGURE E5.**
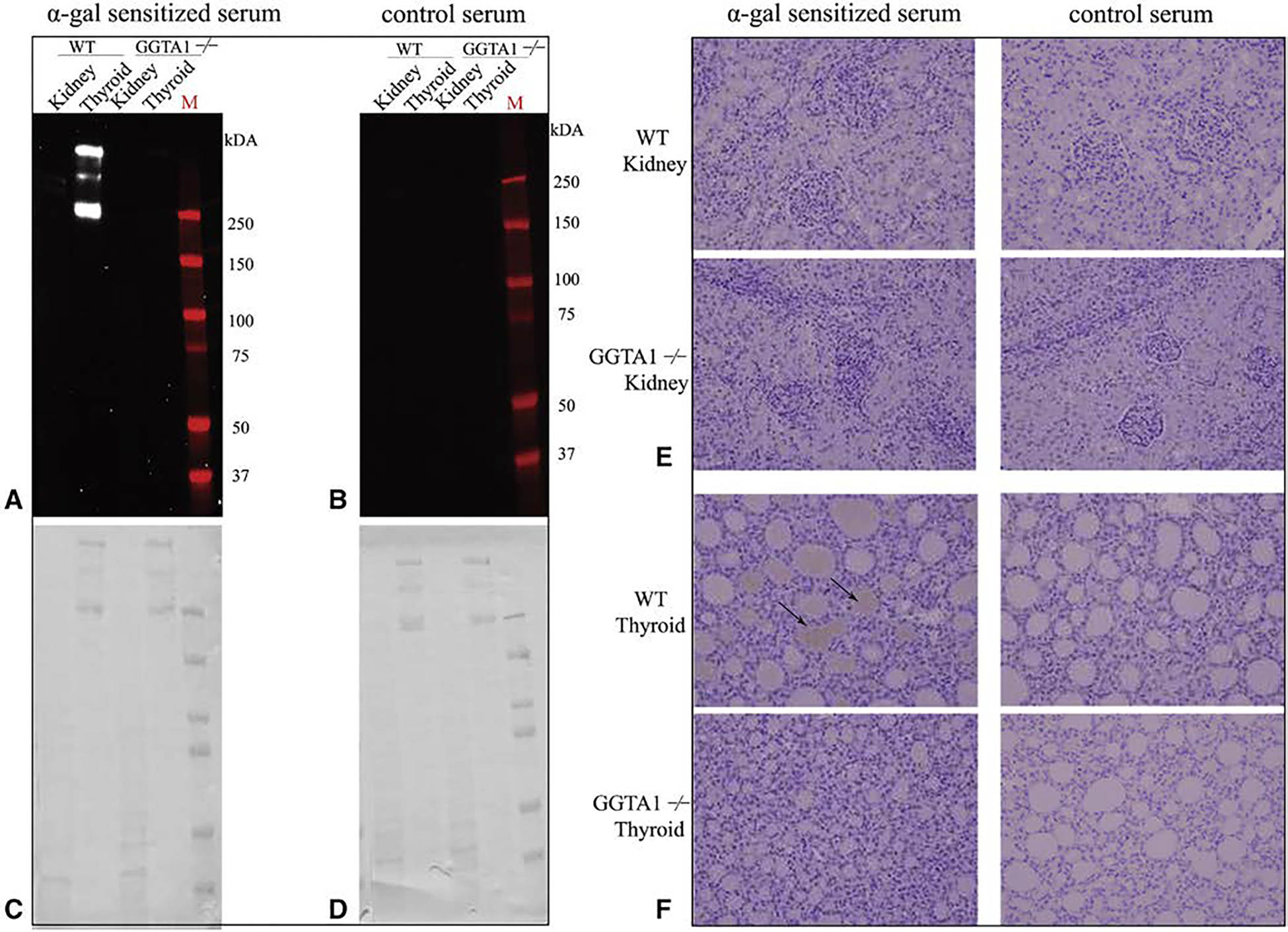
Alpha-gal immunoglobulin E (IgE) reactivity toward different wild type (WT) and glycoprotein galactosyltransferase *α*-1,3 (GGTA^−/−^) porcine organs in a sensitized patient. A, Sensitized patient serum binding profiles for WT and GGTA1^−/−^ kidney and thyroid. B, Alpha-gal binding profiles of WT and GGTA1^−/−^ kidney and thyroid using control serum from patients without elevated alpha-gal IgE. C and D, The Coomassie stains associated with the corresponding immunoblots. E and F, Immunohistochemical binding profile of alpha-gal glycosylated proteins in WT and GGTA^−/−^ kidney and thyroid using sensitized and control serum. Brown staining represents alpha-gal IgE binding. All images are at 200×.

Protein extracts prepared from WT and GGTA1^−/−^ heart, lung, liver, pancreas, muscle, thyroid, and kidney were subjected to 3 different AGS sera types (AGS, sensitized, and control) by immunoblot analysis. AGS patient sera showed positive reactivity to all WT samples (Figures E3 and E4). Six to 8 protein bands were recognized in AGS patient sera staining kidney, heart, and lung tissue extracts (Figure E4). Limited testing of the sensitized patient’s serum demonstrated reactivity to WT thyroid, but not kidney (Figure E5). Control serum did not react to WT samples, and no serum reacted to GGTA1^−/−^ samples (Figures E3–E5).

Taken together, these data confirm that IgE from patients with AGS, and to a lesser degree sensitized patients, interact with WT porcine tissue proteins, but not with GGTA1^−/−^ proteins.

### Alpha-Gal Glycosylation and Human IgE Binding to Commercial Drugs and Implants

**FIGURE 1.**
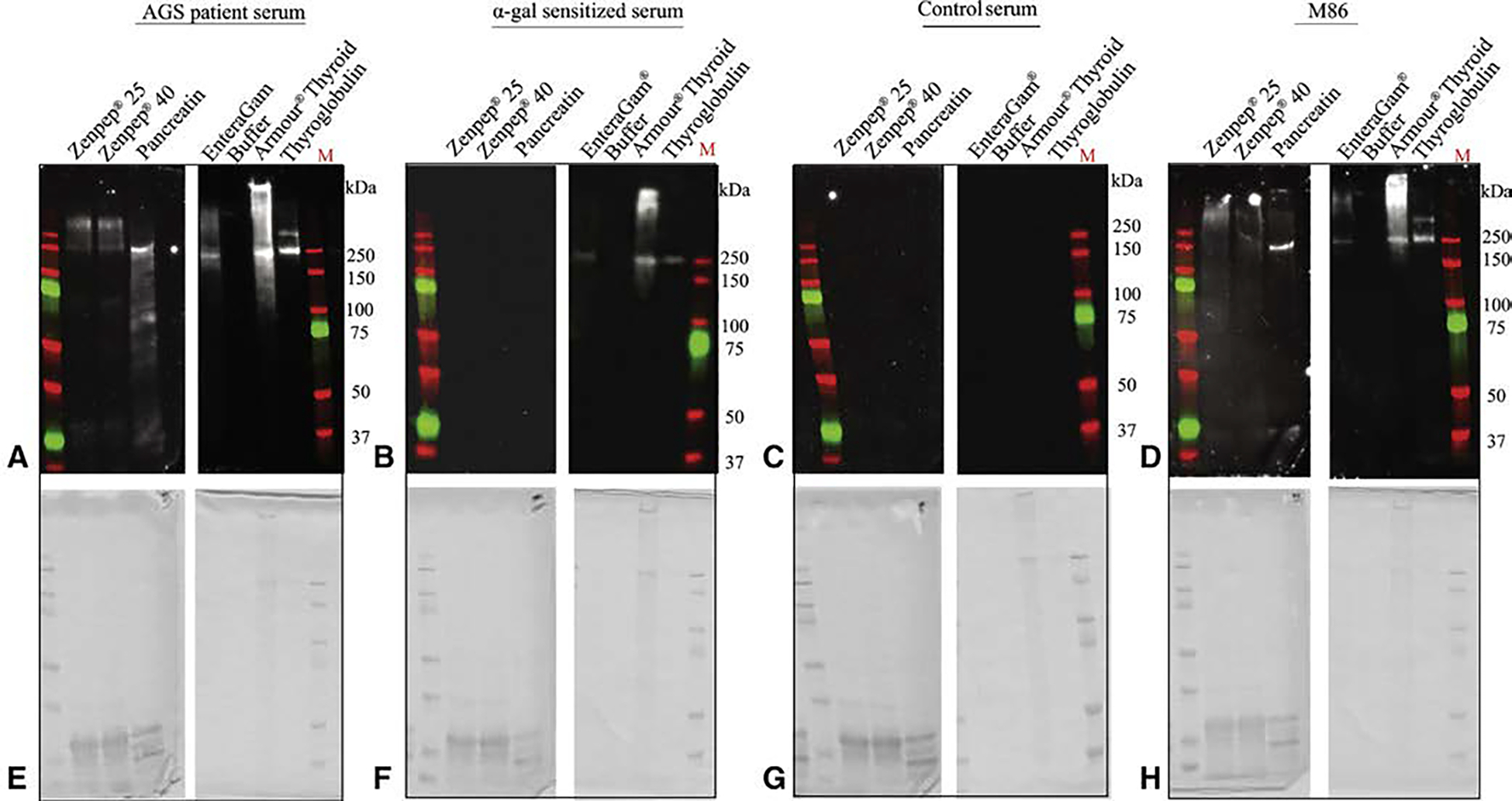
Alpha-gal immunoglobulin E (IgE) reactivity toward commercially available drugs. Immunoblot analysis for alpha-gal IgE reactivity toward Zenpep (Allergan, Irvine, Calif), EnteraGam (Entera Health, Cary, NC), Armour Thyroid (Allergan), and thyroglobulin. A, Using alpha-gal syndrome (AGS) patient serum. B, Using alpha-gal sensitized serum. C, Using control serum from volunteers without AGS. D, Using M86, an alpha-gal antibody. E through H, The associated Coomassie stains indicating the presence of protein.

Alpha-gal was detected in all of the commercially available medical products tested, as demonstrated by M86 immunblot banding (Figure 1). AGS patient sera reacted similarly to M86 in all specimens. EnteraGam and Armour Thyroid also interacted with sensitized patient sera, showing similar 250 kD bands like AGS and M86. Although sensitized patient sera did not react with Zenpep or Pancreatin. Control sera did not react with any of the drugs tested.

**FIGURE 2.**
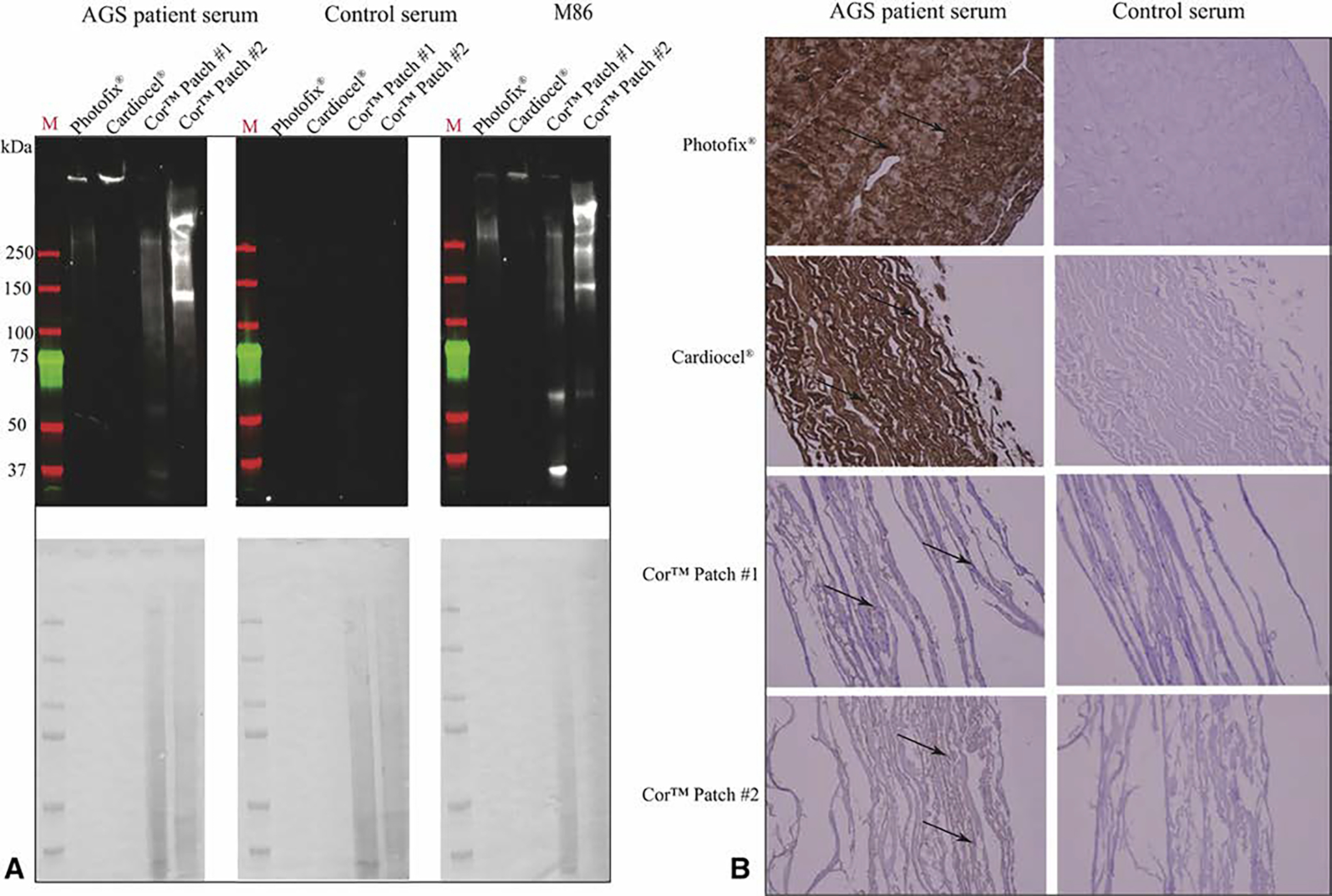
Alpha-gal immunoglobulin E (IgE) reactivity toward commercially available cardiac patch materials. Materials used include Photofix (Cryolife, Kennesaw, Ga), Cardiocel (LeMaitre Vascular, Burlington, Mass), CorMatrix Cor Patch 1.0, and CorMatrix Cor Patch 2.0 (CorMatrix Cardiovascular Inc, Sunnyvale, Calif). A, Immunoblots using alpha-gal syndrome (AGS) patient serum, M86 (an alpha-gal antibody), and control serum from patients without AGS. Assays using serum were counterstained for human IgE B, Immunohistochemisty of the given products using serum from patients with and without AGS. All images are at 200×. Brown staining is indicative of alpha-gal IgE.

**FIGURE 3.**
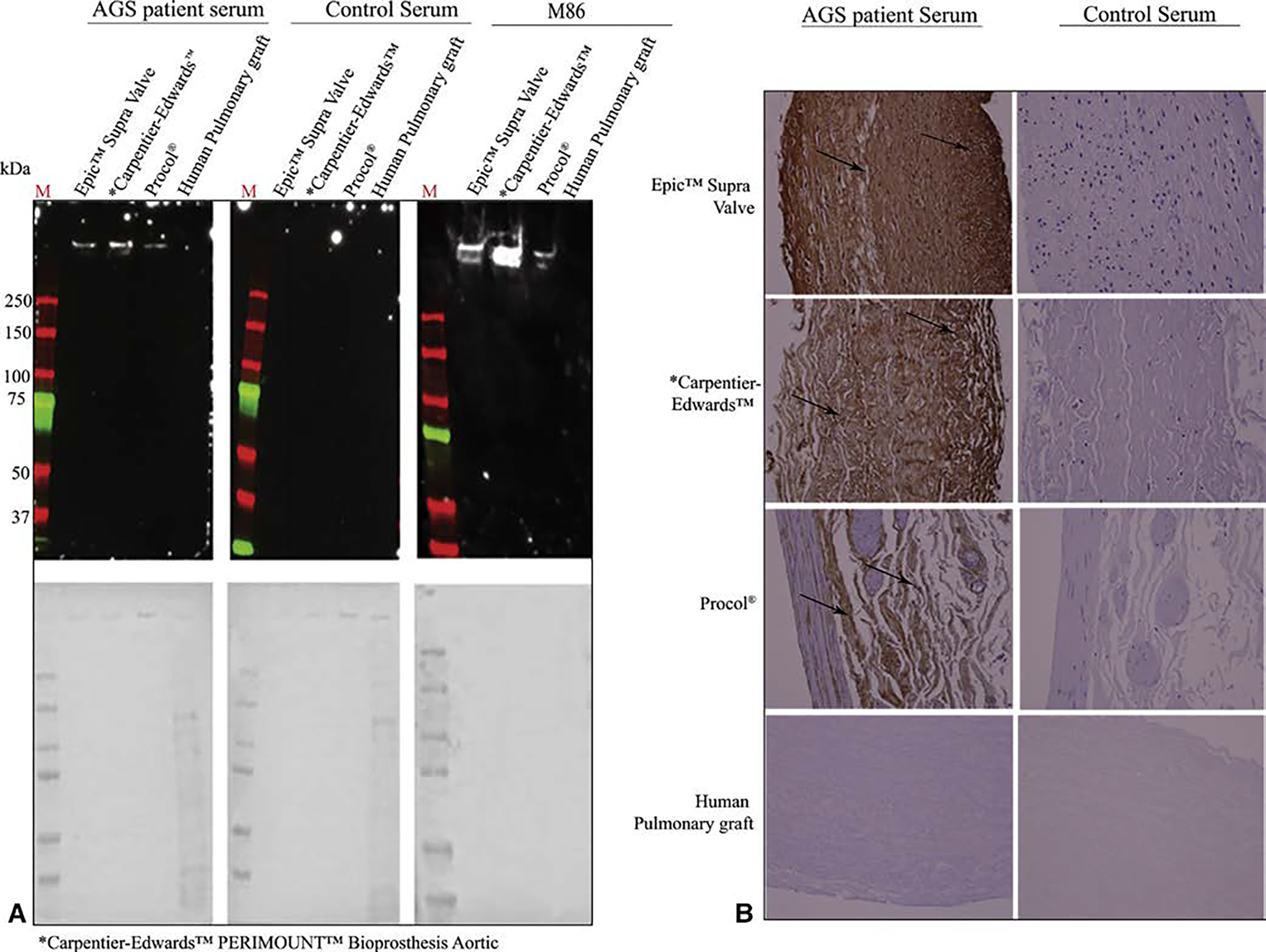
Alpha-gal immunoglobulin E (IgE) reactivity toward commercially available bioprosthetic aortic valves and vascular grafts. Materials used include the Epic Supra Valve (Abbott, Abbott Park, Ill), Carpentier-Edwards Perimount Valve (Edwards Lifesciences, Irvine, Calif), Procol Bovine Mesenteric Vein Conduit (LeMaitre Vascular, Burlington, Mass), and CryoValve SG Human Pulmonary Valve Conduit (Cryolife, Kennesaw, Ga). A, Immunoblots using alpha-gal syndrome (AGS) patient serum, M86 (an alpha-gal antibody), and control serum from patients without AGS. Assays using serum were counterstained for human IgE B, Immunohistochemisty of the given products using serum from patients with and without AGS. All images are at 200×. Brown staining is indicative of alpha-gal IgE.

**FIGURE 4.**
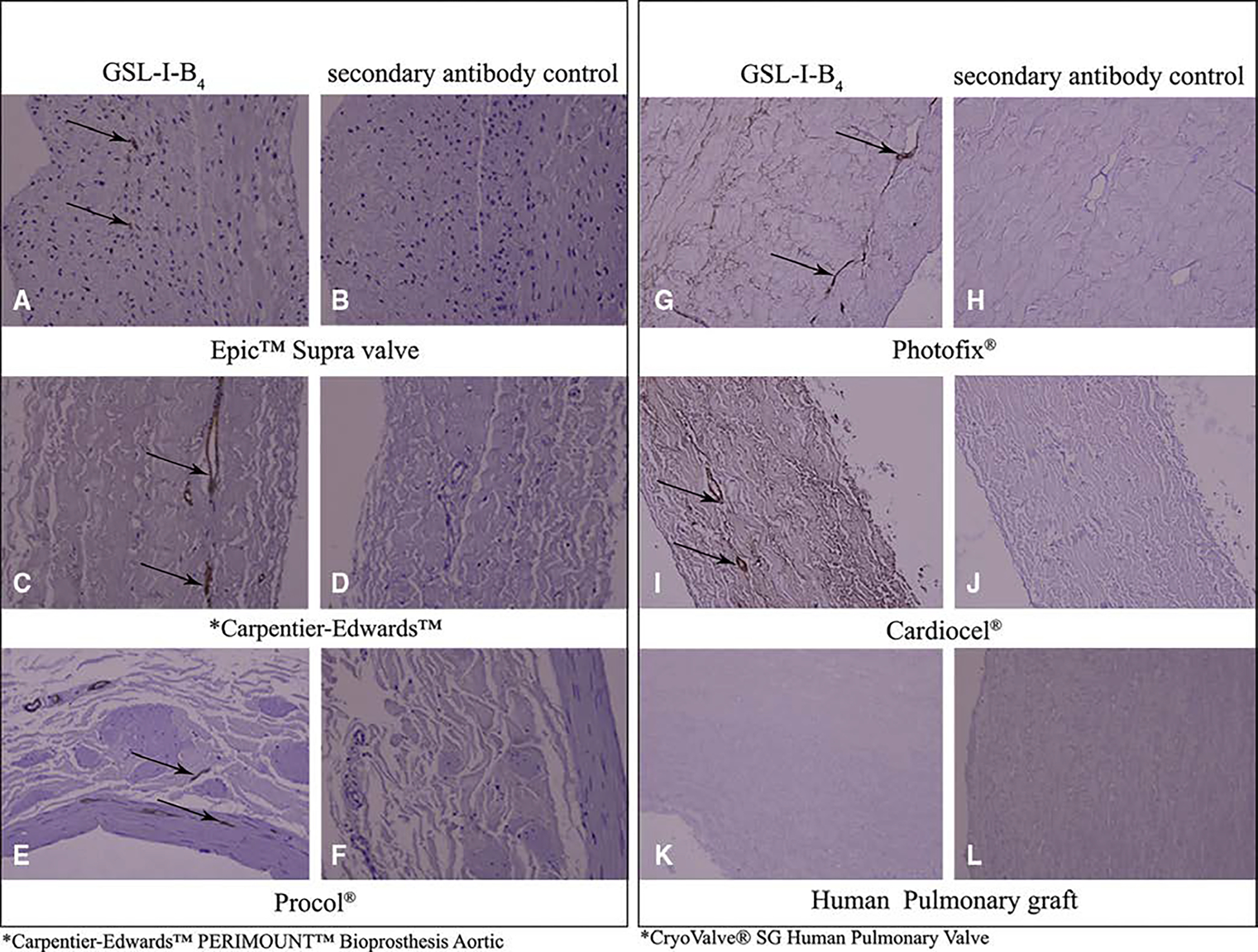
Immunohistochemistry for alpha-gal using commercially available surgical implants. Labels for an associated product are located below the panel. Products included in the figure include Epic Supra Valve (Abbott Park, Ill), Carpentier-Edwards Perimount Valve (Edwards Lifesciences, Irvine, Calif), Procol Bovine Mesenteric Vein Conduit (LeMaitre Vascular, Burlington, Mass), Photofix (Cryolife, Kennesaw, Ga), Cardiocel (LeMaitre Vascular), and CryoValve SG Human Pulmonary Valve Conduit (Cryolife). A, C, E, G, and I, Brown staining (*black arrows*) indicates the presence of alpha-gal using griffonia simplicifolia lectin I – isolectin B4 (GSL I-B_4_), which binds alpha-galactose residues. B, D, F, H, J, K, and L, No staining is seen with the omission of GSL I-B_4_ and use of secondary antibody alone, or in the CryoValve SG Human Pulmonary Valve Conduit, which serves as a negative control. All images at 200×.

AGS and M86 demonstrate similar banding profiles for the included animal-derived surgical implants (Figures 2 and 3). Briefly, AGS and M86 shared similar bands >250 kD across all materials, and in the case of the CorPatch, bands at 30 kD, 60 kD, and 150 kD. Similar bands were not detected in the human pulmonary artery graft controls or with control serum. Immunohistochemistry studies mirrored the immunoblot findings, although of note AGS sera demonstrates a profoundly broader interaction with the surgical implants than the GLS I-B_4_ control antibody (Figure 4).

### Alpha-Gal Glycosylation and Human IgE Binding to Gelatin

**FIGURE 5.**
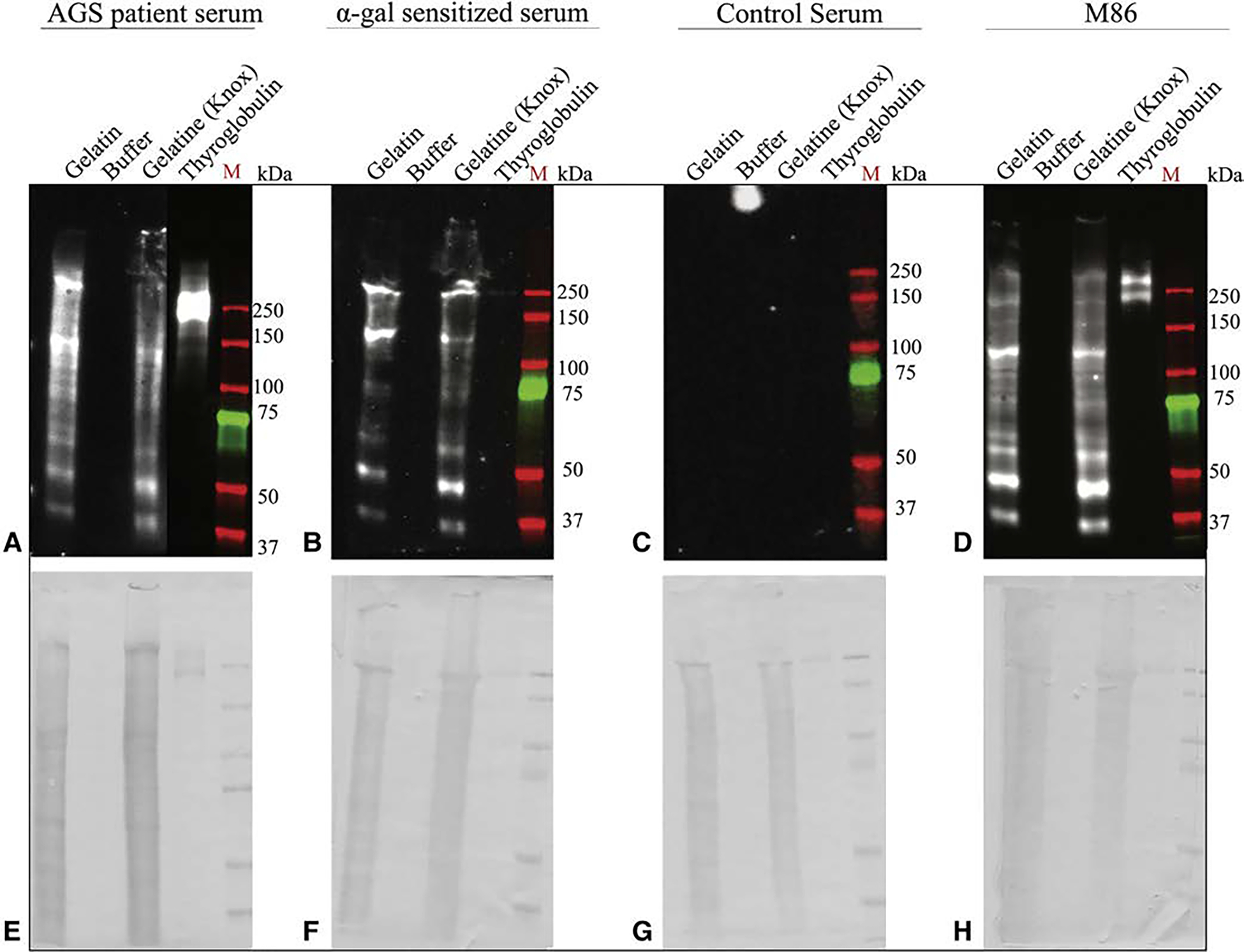
Alpha-gal immunoglobulin E (IgE) reactivity toward commercially available gelatin. Immunoblot analysis for alpha-gal IgE reactivity using laboratory grade gelatin and commercial grade Gelatine (Knox Company, Parsipanny, NJ). A, Alpha-gal syndrome (AGS) patient serum, B, Alpha-gal sensitized serum. C, Control serum from volunteers without AGS. D, M86, an alpha-gal antibody. E though H, The associated Coomassie stains indicating the presence of protein.

Alpha-gal continues to be detected in commercial gelatin products and demonstrates a similar cross reactivity based upon AGS and sensitized patient sera (Figure 5). Briefly, M86 antibodies identify 3 strong alpha-gal bands in gelatin (1 between 100 kD and 150 kD and 2 between 37 kD and 50 kD). Similar banding patterns are seen with AGS and sensitized patient serum. Thyroglobulin again served as a positive control and demonstrated strong AGS and M86 binding, although weaker sensitized sera binding at 250 kD. No reactivity was seen across specimens with the serum from control patients.

## DISCUSSION

AGS is antibody-mediated allergic reaction to the alpha-gal antigen.^3^ Alpha-gal is a common antigen unique to all lesser mammals, and universally leads to the development of IgA, IgM, and IgG antibodies in humans and old-world primates.^1,2^ Recently, tick bites have been associated with IgE seroconversion events and a range of clinical symptoms with future alpha-gal exposure, including red meat.^4–7^ The average duration of this hypersensitivity reaction is unknown, although it is likely chronic given the average time from symptom onset to diagnosis is 7 years.^13–16^ Furthermore, if tick bites are the basis for alpha-gal IgE seroconversion, then patients who experience a single sensitizing episode are more likely to experience future episodes—controlling for geographic region and baseline activity level. Future tick bites, like a vaccine, would heighten or renew patients sensitization to alpha-gal. Although small reports describing the clinical symptoms of AGS exist, the underlying physiological effects and long-term cardiovascular risks remain unknown.^8,29–32^ Herein we demonstrate that alpha-gal continues to be present in commercially available drugs and surgical implants, and has a high affinity for alpha-gal IgE in patients with AGS.

Similar to previously reported studies, we show that alpha-gal continues to be present in Zenpep, Pancreatin, Armour Thyroid, and EnteraGam.^34,35^ Furthermore, these all interact with AGS serum and may lead to adverse reactions clinically. This is important because medications are harder to selectively avoid and symptoms may be inappropriately attributed to patients’ underlying medical issue (eg, pancreatitis, inflammatory bowel, or hypothyroid).

**FIGURE 6.**
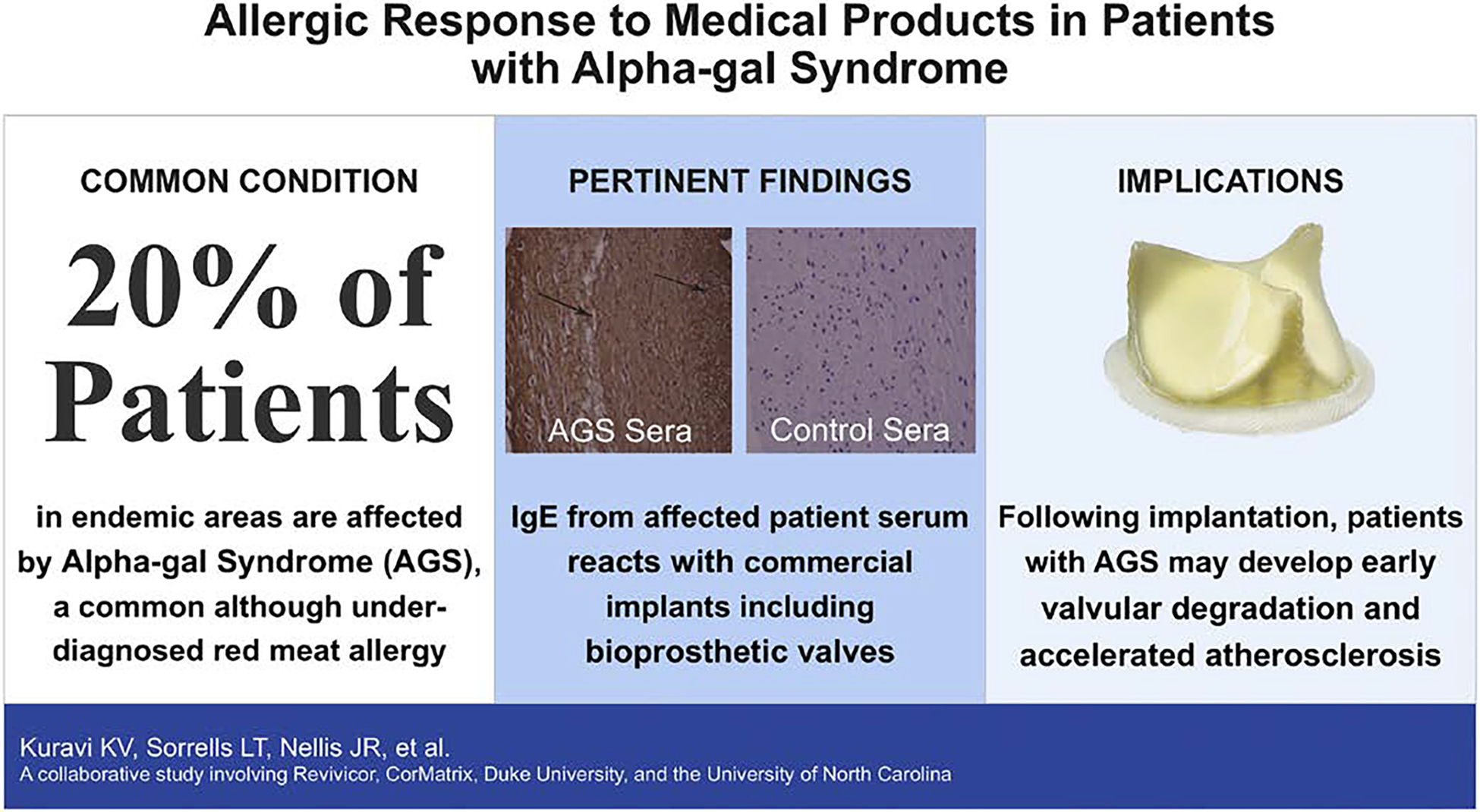
In summary, alpha-gal syndrome (AGS) is a common red meat allergy. Sensitized patients develop an immunoglobulin E (IgE) response to alpha-gal, a carbohydrate that is expressed by all lesser mammals, including cows and pigs. This is particularly important for patients undergoing bioprosthetic valve replacement. As shown, patients with AGS react to the bioprosthetic valve (brown alpha-gal IgE staining based on patient sera), whereas healthy control sera does not (*right panel*). The consequences of this may include chronic inflammation, early valve degradation, and accelerated atherosclerosis.

In addition to drugs, we also show that alpha-gal continues to be present on commercially available bioprosthetic vascular grafts, patch materials, and valves. We do not show the relative immunogenicity of alpha-gal IgM or IgG as a control, but the interaction of alpha-gal IgE from AGS patients with these products is without question (Figure 6). Clinical evidence of AGS patients adversely reacting to bovine or porcine-derived tissues, including heart valves has been reported.^29–31,36^ Hawkins and colleagues^29^ described 2 patients whose valves acutely failed several years after valve replacement following alpha-gal seroconversion. Although our results support a strong immune response in patients with AGS to common cardiac materials, and case reports exist purporting a causal relationship, the clinical evidence of chronic inflammation is lacking.^29,30,37^

Based on our understanding of AGS, a majority of patients experience vague symptoms. When these symptoms are from food, they pass, and the immune response is transient. The results of our study suggest that patients with AGS who undergo cardiac or vascular surgery with animal-derived materials experience an IgE immune response—and because the material is in permanent contact with the blood—this immune response is ongoing. The consequences of this inflammation are likely delayed and subclinical in nature, given the lack of findings in the literature. Although it is worth investigating, given the findings of this study and the growing utilization of bioprosthetic valves and transcatheter aortic valve replacement options. Chronic inflammation in this patient population may contribute to early valve failure and coronary artery disease—conditions that may have otherwise been attributed to patients’ preexisting comorbidities.^8,29^

Despite the findings of our study, the clinical implications of AGS in cardiac surgery are not known. Future studies of particular interest include an outcomes investigation and cohort sampling to determine the association between alpha-gal IgE, early valve degradation, and accelerated coronary artery disease following bioprosthetic valve replacement; prospective preoperative alpha-gal IgE testing to discover the prevalence of AGS for patients undergoing surgery; and animal studies comparing decellularized WT and GGTA1^−/−^ porcine valve function in nonhuman primates before and after alpha-gal IgE sensitization through variable tick toxin exposure. These studies are currently underway.

Lastly, we again demonstrate that GGTA1^−/−^ pig-derived valves represent a commercially available alternative to WT bioprosthetic valves. GGTA1^−/−^ valves and alternative decellularization processes have been suggested before, in response to baseline IgM and IgG alpha-gal mediated immunogenicity and valve degradation.^25–28^ We show that GGTA1^−/−^ pigs did not demonstrate antigenicity using standard alpha-gal antibodies and AGS sera in our study. GGTA1^−/−^ bioprosthetic valves would not only benefit patients with AGS, but also those without AGS.^28^ Furthermore, we show that gelatin, despite among the harshest conditioning processes, continues to carry alpha-gal. This suggests that GGTA1^−/−^ valves may be an easier way toward less immunogenic valves than alternative decellularization or fixation processes.

## CONCLUSIONS

Despite commercial decellularization processes, alpha-gal continues to be present in animal-derived medical products, including bioprosthetic valves. Serum from patients with AGS demonstrates a strong affinity for these products in vitro. This may have serious potential implications for sensitized patients undergoing cardiac surgery, including early valve failure and accelerated coronary artery disease. If these clinical associations are true, GGTA1^−/−^ animals may provide a suitable model from which alternative materials might be made.

## CENTRAL MESSAGE

Alpha-gal is present in animal-derived surgical implants, including bioprosthetic valves. IgE sensitized patients mount an immune response against these materials, which may be detrimental.

## PERSPECTIVE

Alpha-gal is a carbohydrate and common allergen present in animal-derived surgical patch materials, including bioprosthetic valves. IgE-sensitized patients develop an immune response to these materials. Continuous exposure may lead to chronic inflammation and accelerate valvular degeneration and coronary artery disease in select patients.

## Abbreviations and Acronyms

alpha-gal: galactose-*α*-1,3-galactose
AGS: alpha-gal syndrome
*GGTA1*: glycoprotein galactosyltransferase *α*-1,3
GSL I-B_4_: *griffonia simplicifolia* lectin I – isolectin B_4_
PBS: phosphate buffered saline
TBST: Tris-buffered saline with Tween
WT: wild type
